# Socio-economic and structural barriers in Newcastle disease vaccines uptake by smallholder women farmers in Southeastern Kenya

**DOI:** 10.1371/journal.pone.0283076

**Published:** 2023-03-16

**Authors:** Kennedy M. Waweru, Dalmas O. Omia, Lucy Kiganane, Obadia Miroro, Judith Chemuliti, Isaac K. Nyamongo, Salome A. Bukachi

**Affiliations:** 1 School of Business and Economics, Cooperative University of Kenya, Nairobi, Kenya; 2 Institute of Anthropology Gender and African Studies, Nairobi, Kenya; 3 School of Cooperatives and Community Development, Business and Economics, Cooperative University of Kenya, Nairobi, Kenya; 4 Biotechnology Research Institute, Kenya Agricultural Research and Livestock Organization, Kikuyu, Kenya; 5 Cooperative Development Research and Innovation Division, Cooperative University of Kenya Nairobi, Kenya; University of Zambia, ZAMBIA

## Abstract

The exploitation of the full benefits of chicken rearing by smallholder farmers in Sub-Saharan (SSA) Africa is often impeded by poultry diseases which is compounded by limited uptake of vaccination. We interrogate the structural and socioeconomic factors associated with vaccine uptake by women farmers in Southeastern Kenya. A mixed methods design with a convergent approach for comparison of quantitative and qualitative findings was adopted. This involved the administration of a cross section survey to 1274 households, conduct of 23 Focus Groups Discussions (FGDs) and 7 Key informant Interviews (KIIs). Chi Square and t-tests were used to identify factors associated with vaccine uptake. Logistics regression analysis was used to identify the influence of the structural and socioeconomic barriers to vaccine uptake. Findings indicate that having knowledge of Newcastle disease (ND) vaccine increases the likelihood of farmers vaccinating their chicken by up to 32.5 times (95% CI [8.46–124.53]) with a 1 unit increase in vaccine knowledge. A farmer’s distance away from the nearest ND vaccine vendor was found to reduce the likelihood of farmers vaccinating their chicken by up to 4% (95% CI ([0.93–1.00]) for every 1-kilometre increase in distance away from the vaccine vendors. Farmers who considered vaccines to be effective in preventing ND were 39 times (95% CI [6.23–239.8]) more likely to use ND vaccines than those that did not consider ND vaccine to effective. We surmise that a comprehensive approach that addresses increased ND vaccine knowledge among smallholder women chicken farmers, proximity of ND vendors, as well as cost holds the potential for regular and increased ND vaccine uptake.

## Introduction

Poultry keeping remains a major livelihood source across Asia, Africa and Latin America [1]. This practice is characterised by small scale poultry farming in both rural and peri-urban areas with chicken being the most preferred type of poultry [2]. Most rural family poultry productive systems are classified as small extensive scavenging for 2–5 adult birds and extensive scavenging for 5–50 adult birds [3]. The average size flock of rural smallholder famers in Kenya for instance is 10 birds [4, 5] which would classified under the extensive scavenging production system with minimal supplemental feeding. The Food and Agricultural Organization (FAO) notes that despite regional differences in family poultry production systems, women generally undertake the day-to-day management of birds often with the assistance of children [3]. This view is supported by empirical and circumstantial evidence [6–8] indicating that rural women and children who spend most of the time in the homestead are the owners and carers of family poultry. This is because chicken farming requires less capital to start and is easy to manage alongside other domestic chores. Consequently, women make crucial management and investment decisions concerning chicken in their households in comparison to any other livestock such as cattle [9]. Despite the dominance of women in chicken production, men still play some roles in different nodes of the chicken value chain, often serving as market middlemen.

In Kenya, chicken farming makes up about 30% of agricultural contribution to the country’s GDP [10, 11] in Niger Delta poultry farming provides 35% of household’s women income [12]. Besides being a crucial contributor to household income, chicken contributes to women’s income in what Kryger et al., refer to as a side hustle alongside other activities within the women’s domain. It plays an important role in improving women’s livelihood through asset creation [13]. Through social networks, in small informal groups and table banking, women contribute, circulate and share information and money with each other. The latter creates room for buying household items as well as acquisition of more chicken or other small ruminants such as goats, sheep, and cattle. Additionally, chicken farming allows women to create social goodwill with their neighbours and members of the community. In many homes, guests are given live birds to take to their homes after a visit, thus reinforcing a sense of social goodwill in the community [14]. In addition, chicken serve as a form of—insurance, hence, in the event of medical, school emergencies or lack of essential services such as clothes, they come in handy [15]. As Terfa et al., aver, chicken farming also plays a significant role in poverty reduction for the rural communities by ensuring income supply [16]. Also, chicken rearing contributes significantly to household food security, while requiring minimum input in terms of housing, supplemental feeding, disease management and control [14]. Further, through the sale of chicken and eggs, women spend part of the proceeds in ensuring diversified diets [15].

Despite these benefits from chicken, diseases have been identified as one of the biggest barriers facing chicken production [16]. Poultry diseases not only affect productivity but also reduce the flock size within a household through mortality [8]. Ultimately, households whose incomes, social status, food, and nutritional security are incumbent upon the chicken rearing incrementally become vulnerable. Among the high priority and economically important chicken diseases is Newcastle Disease (ND). ND is an infectious viral disease that causes high mortality rates among birds. The disease has no treatment but can be prevented through administration of vaccines. Despite the relative significance of chicken, production in rural Kenya is often affected by diseases with Newcastle disease accounting for 80% of infections. Frequent ND outbreaks suggest endemicity of the disease in Kenya [17, 18]. The mortality rate for unvaccinated flocks can be as high as 100% [18, 19]. Vaccination remains the only available solution for ND however, the availability of vaccines does not translate to increased vaccination rates especially for poultry outside commercial farming [9, 16, 20].

The productive potential of chicken has been associated with the access and quality of animal health services including vaccination uptake [20]. However, a major limitation to vaccine uptake is the profile of most rural smallholder chicken farmers who constitute women smallholder farmers(WSHF) with little formal education [14]. Therefore, many WSHF are not acquainted with facts regarding poultry diseases, causation, and transmission. For instance, a 2017 study across Tanzania, Nepal and India established that 50% of households studied did not know diseases killing their poultry while a quarter of the respondents did not know what they were vaccinating against [21].

Within the rural areas where smallholder chicken production occurs, a myriad of barriers exists these include low access to agricultural extension officers, low investment in technology, limited access to credit and training facilities for farmers, and marginalisation from modern farming techniques. This has seen most WSHF rely on local knowledge for production. Whereas the vaccines that help with disease prevention are available, they are least taken up by WSHF [9, 20] In Southeastern Kenya, the County Government of Makueni and a couple other institutions have been supporting indigenous poultry development [4]. Some of the initiatives include provision of free chicken to households, training on chicken husbandry, feeds & feeding, poultry housing, marketing, group formation, and access to inputs. ND vaccination rates in the county have however remained low estimated at 15% with WSHF continuing to experience high mortality of chicken through ND [22]. It has been suggested that ND control in small scale production systems can enable WSHF’ incomes to be doubled and nutrient intake to improve [23].

Using data drawn from a cross-sectional survey, we interrogate the structural and socio-economic barriers to vaccination uptake faced by WSHF in Southeastern Kenya. Structural barriers often relate to availability, number, concentration, location, transportation, and organizational configuration of ND vaccines service providers [24, 25]. We take cognisance of the fact that, vaccination of chicken with effective and safe vaccines, depends on accessibility, herein examined as commercially distributed safe vaccines within the market, the presence of vaccine supply nodes (agrovets, community vaccinators); the proximity of chicken farmers to supply nodes and, knowledge, defined by women smallholder farmers’ awareness of the ND vaccines and their attendant effectiveness on prevention of ND. These barriers act independently or concurrently with socio-economic barriers.

## Materials and methods

### Background and context of the study

This study was part of an ongoing Gender Inclusive Vaccine Ecosystem (GIVE) (https://idl-bnc-idrc.dspacedirect.org/handle/10625/59232?show=full) action research project being implemented by the University of Nairobi (UON), Kenya Agricultural and Livestock Research Organization (KALRO) and the Cooperative University of Kenya (CUK). The project seeks to enhance the distribution and delivery systems for Newcastle disease and contagious caprine pleuropneumonia vaccines among smallholder farmers as well as increasing women participation in the vaccine value chain in Southeastern region of Kenya.

### Study sites

**Fig 1:**
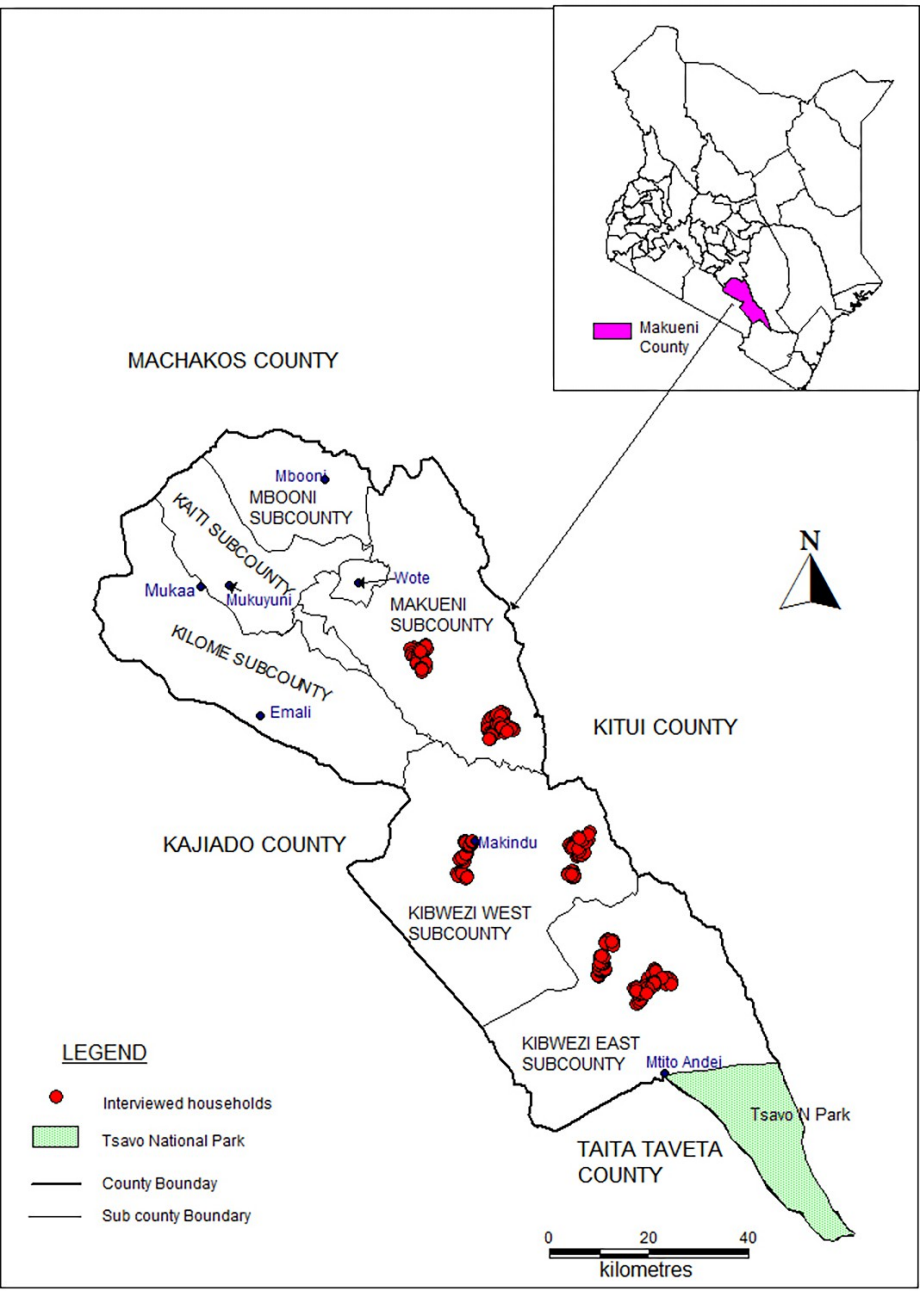
A map showing study area with GPS points of households surveyed.

The study was conducted in Makueni County-Kenya. The County is situated in the Southeastern part of the Country between Latitude 1° 35´ and 3° 00´ South and Longitude 37° 10´ and 38° 30´ East [4]. It covers an area of 8,169.8 Square Kilometres with a population of 987,653. It has a total of 244,669 households each having an average of 4 members with a population density of 121 people [11]. Makueni County is largely (87%) arid more so in the mid and lower parts. The reported average indigenous chicken holdings per household for makueni county is 10 birds [11]. For purposes of this study, three lower lying administrative Sub-Counties: Makueni, Kibwezi West and Kibwezi East were purposefully selected in consultation with Makueni County Department of Agriculture due to the higher prevalence of small-scale chicken faming households. In each of the selected Sub-Counties, two administrative wards were randomly selected namely: Kathonzweni and Kitise Wards in Makueni Sub-County; Kikumbulyu and Makindu Wards in Kibwezi West Sub-County; and Masongaleni, Mtito Andei Wards in Kibwezi East Sub-County. The map of the study site with sampling locations is presented in Fig 1.

### Study design

We employed a mixed methods approach involving household survey, focus group discussions (FGDs) and Key Informant Interviews (KIIs). For the household survey, we sampled 1,274 households across the six Wards. The target households were required to be smallholder chicken farmers. The selection of households followed the adaptive cluster sampling approach with the initial village section being done at random. Neighbourhood villages were added until the target sample size for the ward was reached. The participants to the focus group discussions were purposively selected on homogeneity criterion (small-holder chicken farmers; who had chicken at the moment or three months prior to the study) from the pre-determined ward clusters. Key Informants were selected for their knowledge of local chicken keeping practices they ranged from Sub-County Agricultural Officers, Community Vaccinators, Private Vets, Animal Health Assistants and Local Chiefs.

The survey was designed to measure the outcome variable, household self-reported use of ND vaccines for prevention of ND in the chicken. The survey captured the predictor variables: household demographic characteristics, socio-economic factors, knowledge, attitudes and practice on vaccination, sources of ND vaccines and distances to vaccine vendors. Twenty-three FGDs (12 male only and 11 female only) were carried out with participants ranging from 6 to 12 in number. In Makueni Sub-County, 9 FGDs were conducted (5 male only and 4 female only), in Kibwezi West Sub-County, 8 FGDs were conducted (4 male only and 4 female only) while in Kibwezi East Sub-County 6 FGDs were conducted (3 male only and 3 female only). We conducted Seven KIIs. The FGDs and KIIs provided deeper insights and perspectives from WSHF on the structural and socio-economic barriers to vaccine uptake and provided an opportunity to further probe the findings from the quantitative survey.

The survey questionnaire and qualitative data collection guides were translated into Akamba language with the former administered as an electronic questionnaire on *ArcGIS Survey123 version 3*.*0*. The administration was conducted by pairs of local research assistants, a male and a female, to eligible household members. Eligible respondents included those aged 18 years and above, currently own chicken or had owned them three months prior to the survey. All respondents were read the statement explaining the purpose, the duration, potential benefits and risks of participating in the study. The respondents were informed of the voluntary nature of their participation, those who participated therefore, provided written consent by signing the printed consent form, a copy of which they retained. The study was conducted between January and February 2020. Ethical clearance for the study was granted Strathmore University Institutional Ethical Review: SU-IERC0523/19 and National Commission for Science, Technology and Innovation: NACOSTI/P/19/207.

### Data analysis

Several socio-economic variables (WSHF’s level of education, gender, age of WSHF, number of chicken kept/flock size, number of chicken deaths due to the most recent ND outbreak, perception of effectiveness of ND, previous experience/incidence of ND and type of chicken kept) and structural variables (distance to the nearest vaccine vendor, vaccine packaging) were examined. The use of any type of ND vaccine by the WSHF was considered as adoption or uptake in the study. Distance was measured using proximity analysis of GPS locations of WSHF and nearest location of vaccine vendors. ND vaccine knowledge measured following Campbell et al., [9] who measure ND knowledge by assessing respondent’s ability to correctly answer to a set of five questions about ND vaccines. Each of the question has a ‘Yes’, ‘No’ and ‘I don’t know’ options. The score is calculated by giving one point for each correct response, and no points for an incorrect or “don’t know” response. The correct responses were divided by 5 to give a proportional knowledge score which ranges from 0 to 1. Packaging on the other hand was operationalized as the aptness with which the vaccine packaging dosses aligns with WSHF needs in terms of their flock size.

Analysis of quantitative data involved first conducting exploratory t-tests for continuous variables and Chi square tests for categorical variables to identify those factors that showed statistically significant association with vaccine uptake. The identified factors were then modelled as predictor variables and vaccine uptake modelled as a function of the predictor variables in a logistic regression model to as to identify statistically significant barriers to vaccine uptake. Vaccine uptake is measured as a binary outcome variable where 1 represents households using ND vaccines and 0 represents households not using ND vaccines. Our predictor variables constitute a combination of continuous (proximity distance, proportional knowledge score, flock size number of chicken lost to ND) variables and binary variables (previous incidence of ND in WSHF’s flock, type of chicken kept & rank of farmers source of income). More formally, the logistic regression of the binary outcome variable **‘y’** on predictor variables ***‘x***_***1***_**, *x***_***2*,**_ ***⋯⋯*, *x***_***k***_***’*** estimates parameter values for ***β***_***0***_**, *β***_***1***_**, *⋯⋯*, *β***_***k***_ via maximum likelihood method of the following equation:

$$p=\frac{exp(\beta_{0}+\beta_{1}x_{1}+\beta_{2}x_{2}+\cdots +\beta_{k}x_{k})}{1+exp(\beta_{0}+\beta_{1}x_{1}+\beta_{2}x_{2}+\cdots +\beta_{k}x_{k})}$$ (1)

Qualitative data, from the FDGs and KIIs data were transcribed, translated from Swahili and Akamba to English, reviewed and coded via NVivo-12 after which a thematic analysis was conducted. We used the convergent approach of mixed methods to compare findings from qualitative and quantitative data sources.

## Results

We first present summary statistics to characterise our study population. Descriptive and inferential results from quantitative data are then presented synergistically with qualitative results.

### Summary statistics

The study sampled 1,274 smallholder farmers in the study region with a median age of 48 years (IQR: 36–62) and median age of household head of 52 years (IQR: 42–66). In terms of their highest education level, 766 (60.1%) had attained primary level of education, 285 (22.4%) had at least secondary level of education and 171 (13.4%) had no education while only 9(0.7) had college education. Farming ranked the major as source of income followed by formal employment.

The average number of chicken kept was 15 chicken with majority of study participants 1177 (92.6%) indicating that they keep indigenous types of chicken and only 27 (2.12%) farm improved local types and 59 (4.64%) keep both local and improved types. Our results show that 956 (81.2%) out of 1,177 farmers that keep local breed experienced ND; 24 (88.9%) out of 27 farmers that keep improved local breeds experienced ND and 53 (89.8%) of 59 farmers that keep both local and improved local breeds experienced ND. Various methods are employed by smallholder farmers prevent an attack of ND on their Chicken, the results show that 732 (58%) use herbal interventions, 189 (15%) vaccinate, 126 (10%) use conventional drugs and 208 (16%) do nothing. The results show that out of the farmers that did not vaccine, 471 (51%) were not aware of the existence of ND vaccines. Among those who vaccinate, 174 (92%) reported the vaccines are effective and 13 (7%) reported they are not effective. Among the households not using vaccines, 49.6% indicated that they were aware of the existence of ND vaccines. The reasons cited by the households that did not vaccinate their chicken, lack of knowledge on ND vaccine, cost of vaccines and keeping fewer chicken to warrant vaccination were the most cited reasons (31.2%, 18.2% and 12% respectively). Measurement of knowledge followed the approach Campbell *et al*., [9], who used a proportional knowledge score on a scale of 0–1. Our findings indicate that the overall average proportional vaccine knowledge for the study region score was 0.2. The results of this study show that there were only 307 (24%) of farmers that had received information/training on chicken farming, majority (n = 255, 83%) of whom reported such information/training were adequate.

### Structural and socio-economic factors associated with vaccine uptake

We first run exploratory t-test (presented in Table 1) and Pearson tests of association (presented in Table 2) on continuous and categorical variables respectively to identify factors that had a significant association with the uptake of vaccines. Findings from independent t-tests of continuous variables and vaccine uptake reveal that there was a significant association between distance of the WSHF from vaccine vendors and vaccination uptake (t_1269_ = 3.42, p < 0.001) The mean distance from vaccine vendors for farmers who vaccinated their flock was 8.18 kilometres compared to 10.13 kilometres from those who did not vaccinate their flock. Our findings also showed that the number of chicken kept by a farmers was associated with vaccine uptake (t_1269_ = -8.962, p < 0.001). At the study was conducted, the average number of chicken kept by farmers who vaccinated their flock was 25 while the average number of chicken kept by farmers not vaccinating their chicken was 15. The average number of chicken sold by the farmers within six months prior to the study was also found to be associated with vaccine uptake (t_1269_ = -5.647, p = 0.001). Farmers who did not vaccinate their flock had sold an average of 5 chicken during the six months before the study was undertaken while those that vaccinated their flock had sold an average of 16 chicken. We also found that knowledge on ND vaccines as measured by proportional knowledge score had a significant association with vaccine uptake (t_1269_ = -20, p = 0.001). The proportional knowledge scores for farmers that vaccinated their flock was 0.5 on a scale of 0 to 1, while for farmers that did not vaccinate their flock the average proportional knowledge score was 0.14. In addition, the average number of chicken deaths during an ND outbreak had a significant association (t_1026_ = -4.22, p = 0.001*)* with WSHF vaccination of their chicken against ND.

**Table 1:** Continuous variables associated with vaccination of chicken.

Variables	Does not vaccinate: Mean (SD)	Vaccinates: Mean (SD)	T-Test (P-Value)
AGE OF WSHF (YEARS)	49.96 (16.28)	47.95 (15.02)	1.5857 (0.1131)
AGE OF HOUSEHOLD HEAD (YEARS)	53.74 (15.65)	52.96 (15.47)	0.6390 (0.5230)
NUMBER OF CHICKEN CURRENTLY KEPT	15.23 (13.49)	24.5 (27.76)	-8.962(<0.001)
NUMBER OF CHICKEN SOLD IN THE LAST 6 MONTHS	5.0(14.4)	15.8(52.8)	-5.647(<0.001)
NUMBER OF CHICKEN LOST BECAUSE OF ND	15.21 (19.34)	23.12 (30.14)	-4.22 (<0.001)
DISTANCE FROM VACCINE VENDORS (KMS)	10.13 (7.27)	8.18 (7.0)	3.4189 (<0.001)
ND VACCINE PROPORTIONAL KNOWLEDGE SCORE	0.14 (0.22)	0.50 (0.26)	-20.10 (<0.001)

**Table 2:** Categorical variables associated with vaccination of chicken.

Variable		Vaccination Status		Chi-Sq	p value^a^
		Does not vaccinate (%)	Vaccinates (%)		
Highest Education level of WSHF	No Education	148 (86.6)	23 (13.4)	48.682***	<0.001
	Primary	672 (87.8)	93 (12.2)		
	Secondary	232 (82)	51 (18)		
	College	28 (65.1)	15 (34.9)		
	University	2 (22.2)	7 (77.8)		
	Total	1082 (85.1)	189 (14.9)		
Highest education level of the HH	No Education	146 (88)	20 (12)	53.331***	<0.001
	Primary	610 (87.9)	84 (12.1)		
	Secondary	273 (85.3)	47 (14.7)		
	College	49 (58.3)	35 (41.7)		
	Total	1078 (85.3)	186 (14.7)		
Vaccination effectiveness in ND prevention	Effective	2 (1.1)	174 (98.9)	1195.6***	<0.001
	Not effective	4 (30.8)	9 (69.2)		
	Don’t know	1065(99.7)	3(0.3)		
	Total	1071(85.2)	186 (14.8)		
Type of chicken kept	Local	1,028 (87.6)	146 (12.4)	72.018***	<0.001
	Improved	17 (58.6)	12 (41.4)		
	Both Local and improved	35 (53.8)	30 (46.2)		
	Total	1,080 (85.2)	188 (14.8)		
Experienced a case of ND in your chicken	Yes	885 (85.1)	155 (14.9)	0.054	0.8200
	No	186 (85.7)	31 (14.3)		
	Total	1071 (85.2)	186 (14.8)		

^**a**^p value estimated using fishers exact test; (%) = percentage

*** denotes 1% significance level

Results from our Pearson tests of association on categorical variables show that among factors that had a statistically significant association with vaccination of chicken include education (χ^2^_4, N = 1271_ = 48.682, p = 0.001). Our findings reveal that though there were few chicken farmers (9) with university education only 2 of them did not vaccinate their flock. More than 80% of farmers with basic education and no formal education did not vaccinate their flock compared with 65% of farmers with college education. We found that perception of effectiveness vaccination in preventing ND was associated with vaccine uptake (χ^2^_1, N = 1257_ = 1195.6, p = 0.001), 99% of farmers who perceived vaccines to be effective in preventing ND vaccinated their flock. Vaccination was also associated with the type of chicken kept (χ^2^_2, N = 1268_ = 72.018, p = 0.001), a higher proportion of farmers keeping improved local breeds chicken (71%) and those keeping a combination of both local and improved breeds of chicken (86%) vaccinated their flock, compared to 14.4% of farmers keeping local breeds only who vaccinated their flock. The age of the farmer and a farmer’s experience of ND on their flock was not found to have a significant association with vaccine uptake.

We then selected factors found to have a significant association with vaccine uptake as predictors of our logistic regression model with vaccine uptake as the response variable (the results are presented in Table 3). We interpret the quantitative results from the logistic model along with perspectives from the qualitative findings the study. A farmers distance away from the nearest ND vaccine vendors was found to have a significant influence on vaccine uptake. Our findings show that the odds ratio (OR) in relation to the distance of WSHF from a vaccine vendor is 0.96 (95% CI ([0.93–1.00]) which means that for every kilometre increase in distance away from the vaccine vendor the odds of WSHF vaccinating their flock reduces by -4%.

**Table 3:** Relationship between vaccination and predictor variables.

Variables	Odds Ratio (OR)	Standard Error (SE)	Confidence Interval (CI)
Distance away from vendor(kms)	0.96**	0.023	0.93–1.00
ND vaccine proportional knowledge score	32.48 ***	22.27	8.46–124.53
Number of chicken currently kept	1.02**	0.007	1.01–1.03
WSHF considers ND vaccination as effective	38.67***	36.00	6.23–239.80
Did not experience ND in chicken	0.95	0.202	0.63–1.44
No. of chicken deaths due to Newcastle disease	1.01***	0.004	1.01–1.02
Ranking of WSHF source of income			
Farming	1.35 **	0.182	1.04–1.76
Formal employment	0.17***	0.109	0.05–0.60
Informal employment	0.23*	0.178	0.05–1.04
Business	0.33	0.230	0.09–1.29
Remittances	19.14*	33.16	0.64–571.0
Highest level of education of the respondent (Reference Category–No Education)			
Primary	0.72	0.24	0.38–1.37
Secondary	0.78	0.29	0.37–1.62
College	1.56	0.79	0.58–4.21
University	9.98 **	1.39	1.30–76.79
Types of chicken kept (Local Types—Reference Category)			
Improved Local Types	4.97***	1.92	2.33–10.62
Both Local types and improved local	6.04***	1.60	3.60–10.13
n	1,259		
Pseudo R2	0.2952		
Prob > chi2	0.0000		

OR = Odds Ratio; SE = Standard Error; CI = Confidence Interval

*P-value significant at 90% CI

**P-value significant at 95% CI

***P-value significant at 99% CI.

This is exemplified by the following statements from our qualitative findings when we explored for more insights into this […] “another reason could be the distances because our areas are quite extensive. The distances are usually big (KII, Female). […] it could be stocked in the nearby agro-vets for us to be able to access it. You have to send the Matatus (public service taxis) to buy and bring it which is very costly. If the matatu delays, the vaccine goes bad and we suffer losses” (FGD, Female).

In tandem with proximity analysis of vaccine vendors who were found to be located in major urban Centers only, the following statements from our qualitative findings point to the challenge of distance in vaccine uptake in the different wards of our study […] “The nearby agro-vets don’t dispense those vaccines; we have to go for them all the way to Wote (County headquarters approximately 30km away) to buy them” (FGD, Female). We found out that while our model did not identify cost as significant predictor of vaccine uptake, our qualitative findings reveal that aggregate cost of accessing the vaccines was increased by distances to vaccine vendors as exemplified by the following statement […] “We usually get the vaccines from Kibwezi Town (the nearest urban center) where you have to take a motorbike taxi for 100 Kenya shillings (approximately 1USD) from here, you buy the vaccine and immediately go back and pay another 100 Kenya shillings”(approximately 1USD) (KII, Female).

Results of our model show that vaccine knowledge is a significant predictor of vaccine uptake. A unit increase in WSHF’s proportional knowledge increases the likelihood that they will vaccinate their flock by 32.5times (95% CI [8.46–124.53]). Qualitative findings underscored the challenge posed by lack of vaccine knowledge even where the farmer is aware of the existence of ND vaccines as highlighted by the statement […] “Again, we do not have the knowledge on how to administer the vaccine ourselves and take this scenario where the seller does not have the time to fully explain the procedures to me. So, if I carry this vaccine and I do not know how to use it, it will be another burden because then I have to look for someone who vaccinates to teach me or do it for me. For this reason, I just give up and say ‘chicken, just die’” (FGD, Female).

We also found out that general awareness about the vaccines and their benefits was a factor in ND vaccine uptake as the following statements indicate […] “I do not vaccinate because I do not know the importance of vaccination. Suppose we could get a person from within here to educate us on the importance of vaccination, then we would be willing to vaccinate our chicken; … We hear that chicken are vaccinated but we do not know when and how it is vaccinated. Personally, I have never seen the vaccine; … I do not vaccinate my chicken because I have never seen anyone trained and offering the vaccination services from around here” (FGD, Male). Again, as with distance from the farmers home to the vaccine vendors, the intersection between knowledge and aggregate vaccination cost became apparent as pointed out our qualitative findings […] “Another thing if you call the service providers to come do the vaccination for you, they will demand 10 Kenya shillings (approx. 10US cents) for every chick and this can amount to even being more expensive than buying the chicks” (FGD, Female). The following statement also provides a different perspective on the issue of aggregate cost of vaccination […] “I want to say the challenge is lack of money to buy that vaccine and money to pay the vaccinator, because most people in this area do not know how to vaccinate” (FGD, Female,).

Our findings show that the level of education of a farmer is also a predictor of ND vaccine uptake. The estimated odds ratio (OR) showed that the probability of vaccinating chicken improved markedly as the education level increased. A WSHF with university education is 10 times (95% CI [1.30–76.79]) more likely to vaccinate her chicken compared with one without formal education. However, a farmer with basic education did not show any significant difference in the likelihood of vaccinating her chicken than a farmer without formal education

We established that a farmer’s primary source of income was also significant predictor of ND vaccine uptake. A farmer whose primary source of income came from farming was 1.4 times 3.8% (95% CI [1.04–1.76]) more likely to vaccinate to her chicken compared to those whose farming is not primary source of income. On the other hand, the odds ratio of a WSHF whose primary source of income came from formal employment was 0.17(95% CI [0.05–0.60]). The WSHF was therefore 83% less likely to vaccinate to her chicken compared to those whose primary source of income did not come from informal employment. The odds ratio of a WSHF whose primary source of came from informal employment was 0.23 (95% CI [0.05–1.04]), indicating that she was 77% less likely to vaccinate her flock compared to a farmer whose primary source of income did not come from informal employment On the other hand, a WSHF who depended on remittances for her livelihood was 19 times (95% CI [0.64 0571]) more likely to vaccinate her flock than one who did not. Our qualitative findings established that the income levels of the farmers are also a factor in in vaccine uptake as the following statement illustrate […] “The first biggest problem is levels of income, you have a lot of needs in your home and even your income cannot sustain them all. You see you will not buy a vaccine for 500 shillings while your child has been sent home from school for the same amount; … Maybe now the cost, because during the dry periods, the little money one has is for buying food and not to buy vaccines (KII Female)”.

The results also show that the type of chicken the farmers kept had a significant influence on ND vaccine uptake. The likelihood of WSHF with improved local types of chicken or a mix of both local and improved types vaccinating their flock was 5 times (95% CI [2.33–10.62]) and 6 times (95% CI [3.60–10.13]) respectively compared with those who kept local types only. WSHF who previously experienced deaths of chicken due to ND had an OR of 1.01 (95% CI [1.01–1.02]) indicating a very small likelihood of increasing vaccine uptake compared to those who had not previously experienced deaths due to ND. Though flock size was not found to be a significant predictor of vaccine uptake, there was a strong consensus from our FGDs that the flock size influenced vaccine uptake to some extent. The following statement from our qualitative findings provided more insights to influence of flock size in vaccine uptake. […] “Those with few chicken do not have a lot of chicken to lose if they do not vaccinate their chicken but those with more chicken have a higher stake” (KII, Female). […] “The number of chicken we keep contributes to us not vaccinating. When we buy the vaccine, it requires you to have 50 chicken and above (packaged in 50 doses), so you wonder if you have 10 chicken, you will buy a vaccine worth that money to vaccinate only 10 chicken and the rest go to waste, so the number of chicken we have makes us rethink about buying the vaccine” (FGD, Female);

A WSHF’s view or perception on the effectiveness of ND vaccines was also found to have a significant influence on uptake of vaccines. Farmers who considered vaccines to be effective in preventing ND were 39 times (95% CI [6.23–239.80]) more likely to use ND vaccines than those that did not consider ND vaccine to effective. Our qualitative finding revealed the views of some farmers the surrounding ND vaccines […] “There are also issues because there is a time, we had a program under the national government and we bought vaccines and the people had their own beliefs, they were saying we are going to kill their chicken. …. So, there are those who refused to vaccinate their chicken because of their own beliefs” (KII, Female).

Our qualitative findings indicated ND vaccines were packaged in quantities that were beyond the flock size of most farmers acted as a barrier to vaccine uptake as exemplified by these statements […] “The vaccines are packaged in inappropriate quantities; the least package size is for fifty chicken and once you open it you have to make use of all of it. Most of us keep less than 50 chicken and hence costly for us to use vaccines. This is why we do not vaccinate” (FGD, Female); […] “I don’t vaccinate because like I was telling you earlier, I keep chicken in small scale thus uneconomical to buy vaccines which are very expensive, and their packaging does not qualify a small-scale farmer”. (FGD, Female).

Most farmers (58%) believe in ethnomedicine for chicken diseases management including ND. Qualitative findings corroborate this as highlighted by the following statements […] “Mostly what we do here is that we use herbal remedies to control chicken diseases such; “Kiluma” (*Aloe Secundiflora*) mixed with chicken’s drinking water, “Uthunga”(*Launaea Cornuta*) and also there are some other powder drugs we buy from the agro-vets which we mix with the chicken drinking water …We also use herbal remedies, we mortar the leaves of a tree called “Muharubaini” (*Azadirachta Indica*) and mix the end-product with the chicken drinking water” (FGD, Male).

## Discussion

We examined structural and social economic factors that influence ND vaccine uptake with the goal of informing the design of approaches for enhancing uptake. Our estimates of the average number of chicken kept of 15 chicken per household is generally in line with reported figures of 10 for Makueni County [11]. Smallholder chicken farmers were also reported to keep similar flock sizes elsewhere of 10 chicken in western Kenya [26] and 11 chicken in Tanzania [9]. Our findings that majority (92%) of the chicken farmers kept indigenous chicken is also in line with figures reported by Kenya Population and Housing Census of 94% [11]. The low vaccination rates observed in the study region mirrored rates reported in Western Kenya [5]. A survey of some regions of Tanzania, Mozambique, and Zambia reported low baseline rates which later significantly improved in follow-up surveys after vaccination campaigns were conducted [23]. Lindahl et al., also find that the households in Kenya and Tanzania that had ever used ND vaccine continued to use it after the first month [27]. Our findings indicate that the low vaccination rates do not stem from low prevalence of ND among farmers in the study region since 88.9% reported recent incidence of ND on their flock and higher mortality rates. The higher incidence of ND has also been observed in regions neighboring our study region with incidence rates of 77.9%, which was attributed to lack of a coordinated and sustained effort by the government to prevent ND for smallholder farmers practicing subsistence farmers [28]. This situation has not changed for over 10 years since a survey of literature in 2010 found that most of the smallholder farmers did not vaccinate their flocks and were not even aware that ND can be controlled through vaccination [29].

Efforts to train and increase awareness and knowledge on ND prevention and control in WSHF have been ongoing in Kenya for some time now [30]. However, the mean proportional knowledge scores of WSHF in the study region was 0.2 on a scale of 0 to 1. The mean proportional knowledge score for WSHF that vaccinated their flock was 0.5 compared to those that did not vaccinate whose score was 0.1 and our findings indicate that knowledge of ND vaccine increases the likelihood of farmers vaccinating their chicken by 32.5 times with a 1 unit increase in the proportional knowledge score. FGD findings from the study revealed that general awareness about the vaccines and their benefits was a factor in ND vaccine uptake. This finding is in line with Lindahl et al., who note that in Kenya, having general knowledge about vaccines was associated with more positive attitudes to ND vaccine, both among the households that had tried the vaccine and those that had not [27]. Specific knowledge on the handling and administration of the vaccine in the absence effective veterinary services or a functional system of trained community vaccinators was however vital in both ensuring vaccines are not mishandled in a manner that makes them ineffective, which in turn affects the farmers perception. Efforts to increase farmers knowledge on vaccines do not seem to produce longer lasting changes because they do not take a holistic approach to the key factors at play in vaccine uptake. Insights gained form our qualitative interviews training WSHF without addressing the availability of community vaccinators with an incentive structure that is affordable to the farmers may not result in sustainable uptake of ND vaccines. This view is in line with [31] who argues that designing an incentive structure that is both affordable to the farmer and to community vaccinators has been suggested as a potential solution to sustainable supply of ND vaccines. A slightly divergent view is presented by Campbell et al., who suggest that low vaccination rates are not due lack of awareness of ND but knowledge of the manifestation of the disease. They found that most of households were aware of ND vaccines, and half the households reported previous use of ND vaccines, but only a quarter of households had vaccinated within the last four months [32].

Perception on the effectiveness of ND vaccines was also found to have a significant influence on uptake of vaccines. We found that WSHF who considered vaccines to be effective in preventing ND were 39 times more likely to use ND vaccines than those that did not consider ND vaccine to effective. Campell et al., also revealed that previous ND vaccination was key driver of willingness to pay for vaccines. Which they interpreted as a household’s satisfaction or indicator that households that tried ND vaccines valued them [32]. Negative attitudes about vaccines may be attributed to possible prior negative experiences [27]. This suggestion is corroborated by findings from our FGDs which for instance mentioned that some villages that had received ND vaccination in the past experienced mortality of all their chicken and that due this experience, some farmers in those villages would not allow their flock to be vaccinated in subsequent vaccination campaigns. Addressing the issue of proper handling of vaccines to avoid negative perceptions about vaccines is therefore an important consideration while addressing barriers to vaccine uptake. This view is supported by Terfa, et. al., who assert that it is important to carefully address all other chicken management issues together with vaccination to reduce chicken mortality which is primary concern for farmers so as to reduce farmers’ negative perception about the vaccine and increase the likelihood for future vaccine technologies adoption [16]. In line with this view, Otiang et al., suggest that the primary barrier to chicken management appear to be at the service level [26].

We also found that the type of chicken the farmers kept had a significant influence on ND vaccine uptake. WSHF who kept improved local types of chicken, or a mix of both local and improved types have a 5 times and 6 times higher likelihood of vaccinating their flock respectively compared with those who kept local types only. Although our quantitative survey found that the number of chicken kept by WSHF had a very small influence on the uptake of vaccination, our qualitative findings revealed that the issues of flock size and vaccine uptake was tied to other factors. For instance, the minimum number of doses sold was for 50 chicken while the average flock size was reported to be 15 and farmers found it uneconomical to buy such quantities for small flock. Another view that was expressed was that as the flock size increased the perception of the magnitude of potential loss increased and the willingness to invest in vaccines also increased. This finding is corroborated by Campell et al, finding that the number of chicken the household owned was positively correlated with previous and recent vaccination by the household. They also found that the likelihood of adoption of ND vaccine increases for each additional chicken added to the household’s flock [9]. This finding could be interpreted that as the WSHF flock size increase, she may begin viewing the chicken rearing activity as an economically important activity and thus the willingness to invest in vaccination to reduce the mortality of the chicken.

WSHF whose primary source of income came farming or remittances from family is more likely to vaccinate her flock than WSHF whose primary source of income came from informal or formal employment. This finding is supported by findings from a willingness to pay study in Tanzania which reported that households with any level of on-farm income had higher willingness to pay than households without on-farm income and off-farm income was not significant in influencing willingness to pay [32]. This could be attributed to the fact that the WSHF activities are largely focussed on the farm.

Our study’s finding indicate that most farmers (58%) believe in ethnomedicine (herbal remedies) for treatment of ND. The most common herbal remedy used is *Aloe secundiflora*. This finding is supported by findings from a region adjacent to the study region [28]. They report that 43.3% of chicken farmers the study region use of herbs to treat ND and observed low vaccination rates at 18%. Campell et al., also found that 45% of households reported using traditional medicine and that the use of traditional medicine was the only variable consistently negatively correlated variable with the likelihood vaccinating in their study [9]. It has been suggested that treating chicken with *Aloe secundiflora* reduces mortality and severity of clinical signs but has no significant effect on antibody levels of chicken inoculated with Newcastle disease [33]. It is this quality of reducing the severity of clinical signs by *Aloe secundiflora* that make it a competing factor with ND vaccination. Our qualitative findings support this argument.

Our findings show that distance of WSHF households away from vaccine vendors is negatively associated with vaccine uptake. A 1-kilometre increase in distance away from the vaccine vendors reduces the likelihood of farmers vaccinating their chicken by 4%. With the mean distance from vaccine vendors for non-vaccinating farmers being 10.13kms, it is an indicator that availability of vaccine vendor in centres not too distant from the WSHF could mitigate this barrier. Our qualitative findings indicate structural factors compounded socio-economic factors for instance distance from vaccine vendors was found to increase the aggregate cost of accessing the vaccines.

Packaging of vaccines in quantities that were beyond the flock size of most farmers was also identified through the FGDs as an important structural barrier to vaccine uptake. This finding is in line with findings in Tanzania which suggest that an example of barriers to vaccine access unique to households with small flocks is vaccines sold in large packages with a minimum of 100 doses [9]. A potential remedy to this barrier may be organizing WSHF in groups or cooperatives to leverage on the benefits of collectives.

## Conclusion

Increased ND vaccine knowledge among smallholder women chicken farmers holds the potential for regular and increased ND vaccine uptake. To be effective, the vaccine supply chain needs to address proximity of ND vendors, cost as well as packaging of vaccines in quantities matching the average flock size via a comprehensive approach. Whereas enhancing vaccine knowledge has a very high potential increasing vaccine uptake, addressing individual factors may not lead to sustainable uptake of ND vaccines. Several factors were found to interact and result in a compounding effect as a result, designing a comprehensive strategy that addresses these factors concurrently holds more promise for a sustainable increase in ND vaccine uptake.
